# A Low Spring Constant Piezoresistive Microcantilever for Biological Reagent Detection

**DOI:** 10.3390/mi11111001

**Published:** 2020-11-12

**Authors:** Yuan Tian, Rui Zhao, Yi Liu, Xiaomei Yu

**Affiliations:** 1 National Key Laboratory of Science and Technology on Micro/Nano Fabrication, Institute of Microelectronics, Peking University, Beijing 100871, China; nisioisin@pku.edu.cn (Y.T.); yiliu1@pku.edu.cn (Y.L.); 2 Science and Technology on Electronic Test and Measurement Laboratory, North University of China, Taiyuan 038507, China; zhaorui@nuc.edu.cn

**Keywords:** polyimide, piezoresistive microcantilever, spring constant, deflection sensitivity, ricin

## Abstract

This paper introduces a piezoresistive microcantilever with a low spring constant. The microcantilever was fabricated with titanium (Ti) as the piezoresistor, a low spring constant polyimide (PI) layer, and a thin silicon oxide (SiO_2_) layer as the top and bottom passive layers, respectively. Excellent mechanical performances with the spring constant of 0.02128 N/m and the deflection sensitivity (∆V/V)/∆z of 1.03 × 10^−7^ nm^−1^ were obtained. The output voltage fluctuation of a Wheatstone bridge, which consists of four piezoresistive microcantilevers, is less than 3 μV@3 V in a phosphate buffered saline (PBS) environment. A microcantilever aptasensor was then developed through functionalizing the microcantilevers with a ricin aptamer probe, and detections on ricin with concentrations of 10, 20, 50 and 100 ng/mL were successfully realized. A good specificity was also confirmed by using bovine serum albumin (BSA) as a blank control. The experiment results show that the Ti and PI-based microcantilever has great prospects for ultrasensitive biochemical molecule detections with high reliability and specificity.

## 1. Introduction

The microcantilever was developed some time ago as a miniature biochemical sensor with high sensitivity. Its readout methods can be classified as optical method [1,2], piezoresistive method [3,4,5], and capacitive method [6,7]. Although traditional optic analytical methods have relatively high sensitivity, they require large, expensive laser instruments and well professional and technical personnel to operate them. Compared to optical analytical methods, a piezoresistive microcantilver-based sensor is easy to use by reading out the signals with high precision multimeter or complementary metal oxide semiconductor (CMOS) circuits. It can also be applied in a liquid environment without sensitivity loss [8].

A single crystalline silicon-based microcantilever fabricated by silicon-on-insulator (SOI) wafer is widely used to realize biochemical detections. In 2017, Zhao el al. reported a Si-based piezoresistive microcantilever that was able to detect Dimethyl methylphosphonate (DMMP) with a sensitivity of 1.0 μM, and the spring constant of the microcantilver was approximately 0.04679 N/m [9]. In 2017, Patkar et al. created a dynamic mode piezoresistive microcantilever with a spring constant of 0.2 N/m, a deflection sensitivity of 0.3 ppm nm^−1^, and a resonant frequency at 22.5 kHz [10]. In 2018, Ku et al. invented a piezoresistive microcantilever using SiO_2_, Si_3_N_4_, and polysilicon, which realized a minimum detection limit of C-reactive protein (CPR) concentration at 100 μg with 3.1 N/m surface stress change by a temperature compensation [11]. Xu et al. introduced a piezoresistive microcantilever with silicon nanopillars and ZnO nanorod on the top of the surface, which achieved an increase of the surface area by a factor of about 100 and a 2.1 ppb detection limit of NO_2_ detection [12,13]. In 2020, Rotake et al. studied a piezoresistive microcantilever for Hg^2+^ detection with a limit of 0.75 ng/mL. The spring constant of the microcantilever was 0.07053 N/m [14]. In 2020, Kandpal et al. reported a piezoresistive microcantilever which realized a 1,3,5-trinitroperhydro-1,3,5-triazine (RDX) detection with 6-MNA surface functionalization. The spring constant was about 0.2 N/m and the deflection sensitivity was approximately 0.161 ppm/nm. RDX vapors were detected within about 30 s exposure time [15].

To fabricate a microcantilever with higher sensitivity, a thin single crystalline silicon layer is required, which can only be obtained from an expensive silicon-on-insulator (SOI) wafer, which is easily broken during applications because of the high spring constant. Flexible piezoresistive microcantilevers were developed with metal as the piezoresistor and a polymer as the passive layer in recent years. In 2011, Zhu et al. studied a V-shaped piezoresistive microcantilever with polyimide as the structural layer. The polyimide (PI)-based microcantilever has 17.5 μm length and 1.45 μm thickness. The deflection sensitivity of the microcantilever is 1.1 × 10^−6^/nm^−1^, and the detection limit of TNB gas is 10 ppb [16]. In 2013, Liu et al. reported a rectangular microcantilever with composite polyimide as the structural layer and 40 nm gold as adhesion layer. The length, width, and thickness of the microcantilever are 1000 μm, 200 μm, and 25 μm, respectively, which achieved the detection of surface stress change of 5.66 N/m caused by YN94 growth [17]. These microcantilevers are relatively thick and the sensitivity is very limited.

In this work, a flexible microcantilever was fabricated on a silicon wafer with Titanium (Ti) as the piezoresistor and thin polyimide (PI) and SiO_2_ as the passive layers. A spring constant of 0.02128 N/m and a deflection sensitivity of 1.03 × 10^−7^ nm^−1^ were successfully achieved.

## 2. Design and Fabrication 

### 2.1. Design of Piezoresistive Microcantilever

Polyimide is widely used in MEMS device for its low residual stress, low coefficient of thermal expansion, low water absorption, and good insulation performance. PI 2610 has a Young’s modulus of 8.5 Gpa. To obtain a low spring constant microcantilever, 1.3 μm thick PI was selected as the top passive layer and a 100 nm thick SiO_2_ as the bottom layer in this work. 

For a piezoresistive microcantilever, the sensitivity is defined as the relative resistance change (Δ*R/R)* versus the free end vertical distance change (Δ*z*), which can be described as [18]:

(1) $$\frac{∆R/R}{∆z}=\frac{{3Kt(l}_{c}{-l}_{leg}/2)}{{2l}_{c}^{3}}\;$$

where *K* is the gauge factor, *t* is the thickness, *l_c_* is the length of the microcantilever, and *l_leg_* is the piezoresistor length. The gauge factor is defined as [19]:

(2) $$K\;=\;\left(1\;+\;2\nu \right)+\frac{∆\rho /\rho }{\varepsilon }\;$$

where *ν* is Poisson’s ratio of the piezoresistor, *ε* is the length change of the piezoresistor, ∆ρ is the electrical resistivity change of the piezoresistor, and *ρ* is the original electrical resistivity of the piezoresistor. To increase the piezoresistive microcantilever’s sensitivity, we must increase the gauge factor and thickness of the microcantilever, and decrease its length. Although single crystalline silicon has a high piezoresistive coefficient, its spring constant and the fabrication cost are higher, since the thin single crystalline silicon layer can only be obtained from an expensive silicon-on-insulator (SOI) wafer. Another type of piezoresistive material is metal. For metal materials, their electrical resistivity is quite stable at the same temperature. ∆ρ/ρ has almost no contribution for ∆R/R. The most contributing parts of the resistance change are Poisson’s ratio and the length change. Most metal materials show a relatively high positive electrical temperature coefficient of resistance (TCR), so a low TCR piezoresistive material is expected to improve the microcantilever stability. Titanium has a minimum TCR of 0.00063 °C^−1^ and medium Poisson’s ratio of 0.34 [20,21] among all metal materials, hence, it was implemented as the piezoresistive material to ensure stable output voltage and high sensitivity in our design. 

The sensitivity of the microcantilever is also closely related to its length and thickness, therefore, it is necessary to carefully optimize the parameters of the microcantilever for it to be able to detect biochemical molecules. To avoid the microcantilever sticking to the substrate and keep a low spring constant at the same time, a rectangular PI/Ti/SiO_2_ piezoresistive microcantilever was designed with a dimension of 100 μm in length, 50 μm in width, and 1.44 μm in thickness. To ensure high sensitivity, the piezoresistor was placed at the root of the microcantilver. Also, the length should be as long as possible to decrease thermal drift at the same time. Thus, a four-fold shape piezoresistor with a single leg dimension of 60 μm × 5 μm × 40 nm was designed. With the above designed parameters, the sensitivity of the microcantilever is calculated to be 3.645 × 10^−7^ nm^−1^ according to Equation (1). 

**Figure 1:**
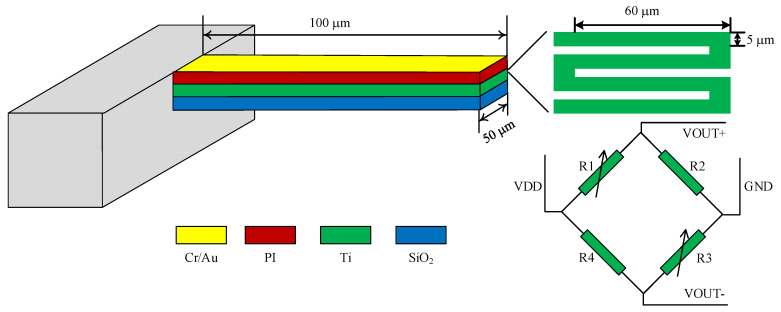
The schematic diagram of the polyimide (PI)/titanium (Ti)/silicon oxide (SiO_2_) microcantilever. The shape of the piezoresistor is four-fold and four microcantilevers consist of a Wheatstone bridge.

As shown in Figure 1, the microcantilever consists of a 1.3 μm thick PI top passive layer, a 40 nm thick titanium piezoresistive layer, and a 100 nm thick SiO_2_ bottom passive layer from top to bottom. To reduce the impact of environmental noise and initial thermal mismatch, four microcantilevers form a sensor set of Wheatstone bridge. The Wheatstone bridge was configured as half-bridge type as shown in Figure 1 [22], in which nonadjacent two were coated with a 10/40 nm Cr/Au modified layer as the sensing microcantilevers, which will bent and the resistance of the piezoresistor will be changed due to the reaction process between the sensing and target molecules, while other two are only with PI on the top as reference microcantilevers. The differential signal output of the Wheatstone bridge *V_out_* can be described as:

(3) $${\;V}_{out}=\frac{1}{2}\;V_{in}\;\frac{∆R}{R}$$

where *V_in_* is the supplied voltage, *R* is the original resistance of the piezoresistor, and Δ*R* is the resistance change of the piezoresistor. According to Equations (1) and (3), the sensitivity of the microcantilever can be finally written as:

(4) $$\frac{\;∆V/V}{∆z}=\frac{{3Kt(l}_{c}{-l}_{leg}/2)}{{4l}_{c}^{3}}\;$$

### 2.2. Fabrication 

**Figure 2:**
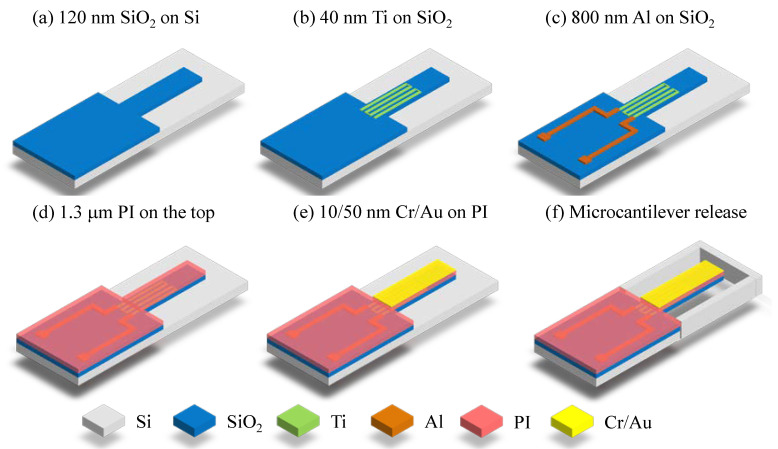
The fabrication processes of PI/Ti/SiO2 microcantilevers. (**a**) LPCVD technology was used to deposit 100 nm SiO_2_. (**b**) Sputtering was used to make 40 nm thick Titanium piezoresistor. (**c**) Sputtering was used to deposit 800 nm thick Al pad. (**d**) Spin coating was implemented to make 1.3 μm thick PI. (**e**) Sputtering was used to deposit 10/50 nmthick Cr/Au modified layer. (**f**) A combination of anisotropic and isotropic dry etching techniques was used to etch the silicon wafer until the microcantilever structure is completely released.

The microcantilever was fabricated on a 525 μm thick N-type (100) single-polished silicon wafer. As shown in Figure 2, the entire fabrication process includes following a 5-step photolithography process:

(a) Firstly, LPCVD technology was used to deposit a 100 nm thick SiO_2_ on the surface of the silicon wafer as the bottom passive layer of the microcantilever. After the first photolithography, BHF was used to remove the SiO_2_ outside the pattern to define the SiO_2_ layer of microcantilever.
(b) Then, a 40 nm thick Titanium was sputtered and patterned to form the Ti piezoresistor.
(c) An 800 nm thick Al was sputtered, and Al connection wires and pads were patterned in the third photolithography.
(d) After that, a 1.3 μm thick PI was spin coated on the surface of the substrate at 3000 rpm for 30 s. The wafer was then annealed in a nitrogen atmosphere with a gas flow of 4.5 L/min and a temperature of 350 °C for 90 min to complete the PI layer curing. Apart from the piezoresistor, the PI layer also fully encapsulates the aluminum wires to avoid electrical conduction in an aqueous environment.
(e) E-beam evaporation was used to deposit a 10/50 nm Cr/Au modified layer on the surface of the PI layer. Cr was used as the adhesion layer between PI and Au. After the fourth photolithography, the Au/Cr was etched by wet etching technology to form a biochemical molecule modified layer in sensing microcantilevers.
(f) At last, the microcantilever was patterned and defined by using a 6 μm thick photoresist as a mask. After PI was etched by anisotropic RIE oxygen plasma etching until the silicon wafer, a dry silicon etching techniques was used to etch the silicon wafer from front side until the microcantilever structure was completely released, and a reactive well was also formed at the same time. The traditional release of the microcantilever structure by the isotropic dry etching technology can cause serious lateral undercutting situation problems at the sidewall near the fixed end of the microcantilever. A combination of anisotropic and isotropic dry etching techniques was used to avoid this lateral undercutting problem of SiO_2_.

**Figure 3:**
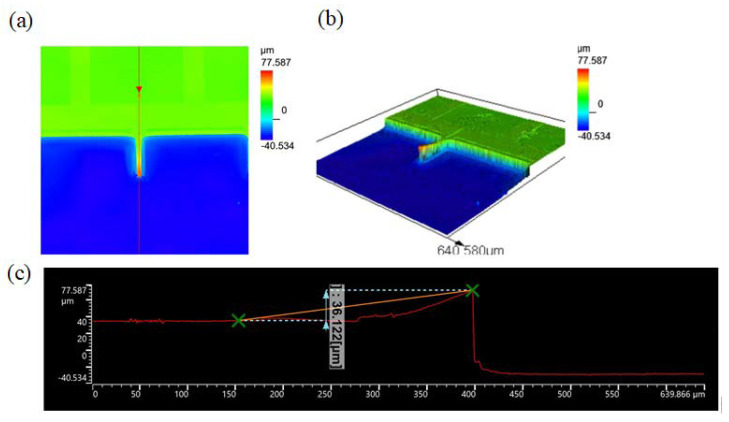
3D measurement and observations images by laser confocal scanning microscope LEXT OLS5000 (**a**,**b**) are the top and 3D visions of a microcantilever. The height of free end is 77.587 μm. (**c**) is the height scope of the microcantilever. The upper bend of the free end of the microcantilever is 36.122 μm and the depth of reactive well is 40.534 μm.

A laser confocal microscope OLS 5000 was introduced to rebuild a 3D vision of the microcantilever, as shown in Figure 3 Due to the optic projection, the hollow space below the microcantilever cannot be well displayed in Figure 3a. As shown in Figure 3b, a deep reactive well fabricated by the anisotropic dry etching can be seen clearly. Figure 3c is the height scope of the microcantilever; it can be seen that the microcantilever bent upward for 36.122 μm and the depth of the reactive well is 40.534 μm. Since the coefficient of thermal expansion for PI is higher than that of Si in this temperature range, the tensile stresses in the PI films [14] made the microcantilever bend upward, which can inhibit the microcantilever from sticking to the bottom of the reactive well.

**Figure 4:**
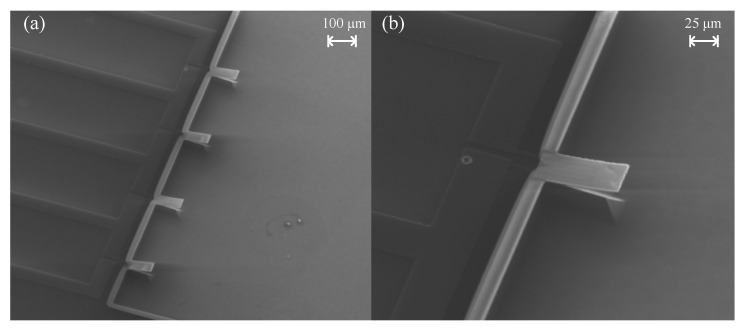
SEM image of (**a**) four microcantilevers that make up the Wheatstone bridge and (**b**) a sensing microcantilever respectively.

A full vision of the microcantilever was also introduced by scanning electronic microscopy (SEM) to get a higher quality structure image. Figure 4a is the SEM image of a Wheatstone bridge with four microcantilevers, and Figure 4b is a sensing microcantilever. 

## 3. Performances

### 3.1. Spring Constant

**Figure 5:**
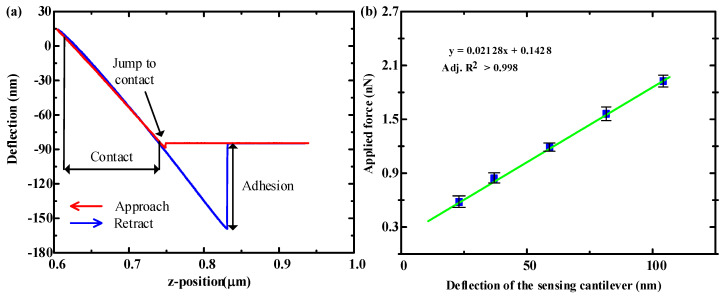
(**a**) The relationship curve between the deflection displacement of the microcantilever and the AFM tip position change under the AFM load force. (**b**) The linear correlation of the relationship between the load force on a microcantilever and its deflection displacement.

It is difficult to calculate the spring constant k for a multi-layers microcantilver, so we measured it with a reference cantilever method. An AFM probe (MFP-3D-BIO PNP-DB) manufactured by Nanoworld in Switzerland was used as a reference microcantilever. The AFM probe has a rectangular structure with a geometric size of 200 μm × 13 μm × 0.6 μm, an intrinsic resonance frequency of 17 kHz, and a spring constant of 0.06 N/m. Figure 5a shows the measured relationship between the deflection of the microcantilever and the displacement of the piezoelectric driven AFM probe under a load. The red curve in the figure represents the approach process of the AFM probe, and the blue curve represents the AFM probe retract process. As the AFM tip approaches the microcantilever surface, initially the forces are too small to give a measurable deflection of the AFM tip. Then the attractive forces (such as Van der Waals) overcome the AFM tip spring constant and the tip jumps into contact with the microcantilever surface. Once the AFM tip contact the microcantilever’s surface, it remains on the surface as the separation between the AFM tip and microcantilever surface, causing a deflection of the AFM tip and the microcantilever. As the AFM tip is retracted from the microcantilever surface, often the AFM tip remains in contact with the surface due to some adhesion and the AFM tip is deflected downwards. At some distance, the force from the AFM tip can finally overcome the adhesion and get to the initial stage [23]. Based on the AFM probe approach process and spring constant of AFM probe, we can establish the relationship between the applied force and the deflection displacement of the microcantilever as shown in the Figure 5b. The spring constant of the microcantilever is obtained to be 0.02128 N/m by the fitted line slope, which is almost half of the one compared with the silicon-based microcantilever of 0.04679 N/m [24]. The adjustment coefficient of the fitting result is adj. R^2^ = 0.998, indicating that the microcantilever has a good linear response for applications of the PI/Ti/SiO_2_ microcantilever 

### 3.2. Sensitivity

**Figure 6:**
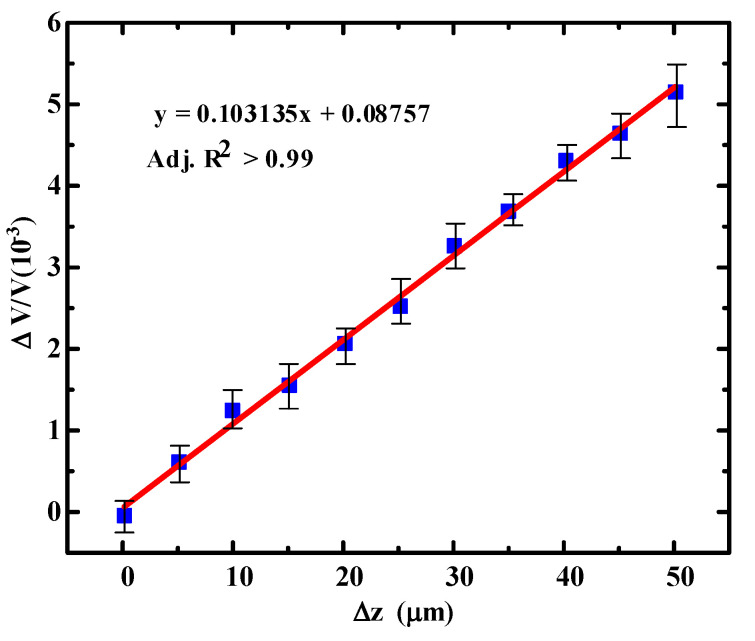
The relationship between the vertical displacement change Δ*z* of the free end of the microcantilever and the relative voltage change Δ*V/V*.

The deflection sensitivity is (∆V/V)/∆z, where Δ*z* is vertical displacement change of free end of the microcantilever. In order to obtain the deflection sensitivity, the microcantilever was fixed on a probe station in which the probe was driven by a precision electronically controlled translation stage with a minimum step size of 5 μm. During the test, the probe put a pressure at the free end of the microcantilever, and thus changed the piezoresistor value. The output voltage of the microcantilever was measured by an Agilent 34,401 A 6^1⁄2^ mustimeters. The relative voltage change (Δ*V/V*) versus the vertical displacement change Δ*z* for the PI/Ti/SiO_2_ piezoresistive microcantilever is shown in Figure 6. By linearly fitting the data between the output voltage change of the microcantilever and the vertical displacement of the microcantilever, a deflection sensitivity of 1.03 × 10^−7^ nm^−^^1^ was obtained. The determination coefficient of the fitting straight line Adj. R^2^ is above 0.99, indicating that the flexible PI/Ti/SiO_2_ microcantilever has a good linear relationship.

### 3.3. Stability

**Figure 7:**
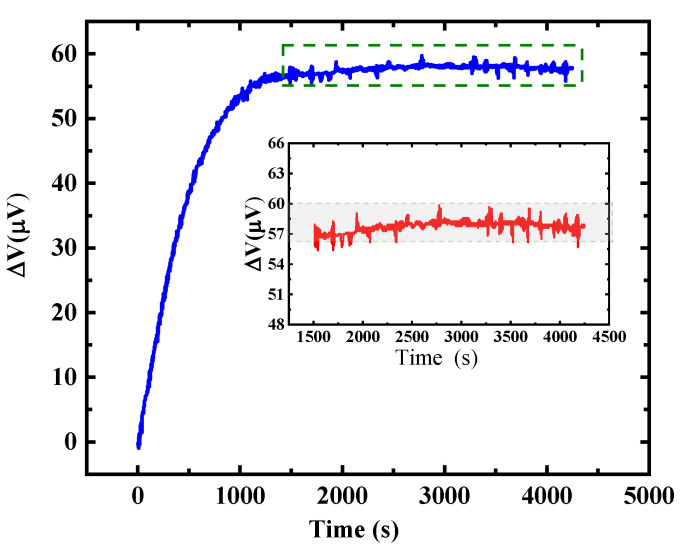
The output voltage fluctuation of a microcantilever in PBS buffer (PH 7.4, 0.01 m) at temperature (20 °C) and 3 V bias voltage. Inset shows the voltage fluctuation is less than 3 μV.

The biochemical detection experiments involved in this work were carried out in a liquid phase environment containing inorganic salts. In order to verify the feasibility of the microcantilever for trace detections, we tested the stability of the sensor output signal in phosphate buffered saline (PBS) with a pH of 7.4 and a concentration of 0.01 M. Firstly, the microcantilever chip was fixed on a printed circuit board (PCB) and electrically connected to the PCB through wire bonding. Then, a 3 V DC bias voltage was applied to the bridge, and the output differential voltage signal was measured by an Agilent 34,401 A 6½ high-precision multimeter, and real-time data acquisition was performed by the upper computer. Figure 7 shows the test results of the stability of the output signal of the microcantilever in the PBS solution. It can be observed from the figure that the output signal of the sensor increases rapidly during the initial period of time (about 20 min), which is caused by the self-heating of the titanium piezoresistor under the bias voltage. Due to the different thermal expansion coefficients of PI and SiO_2_, the microcantilevers have different thermal deformations, which ultimately lead to the output signal changing in the initial stage. Subsequently, the microcantilever output signal reached a state of dynamic equilibrium. The temperature cross sensitivity played a minor role during the detection, while the 1/f noise and Johnson noise of the piezoresistors played a leading role. The fluctuation range of its output voltage was less than 3 μV as shown in the inset of Figure 7, and this experimental results show that the microcantilever prepared in this paper has low output voltage noise in PBS solution, and can meet the demand for the trace level detections of biochemical molecules.

## 4. Biological Detections

### 4.1. IgG Detections

As a regular biosensor detection indicator [25,26,27,28], IgG was first introduced to evaluate the detection sensitivity of the microcantilever. Related experiment materials of 3,3’-Dithiopropionic Acid (DDPA), 1-Ethyl-3-(3-dimethylaminopropyl) Carbodiimide Hydrochloride (EDC), N-Hydroxy Succinimide (NHS), Streptavidin, Ethanolamine, bovine serum albumin (BSA), and PBS solutions were all purchased from Sigma-Aldrich (Shanghai, China). Biotinylated goat anti-human IgG (H+L) polyclonal antibody was purchased from Bioss Antibodies Inc. (Beijing, China). Concentrated sulfuric acid (H_2_SO_4_, ≥95–98%), hydrogen peroxide (H_2_O_2_, ≥30%), and concentrated hydrochloric acid (HCl, ≥38%) were purchased from Beijing Chemical Works (Beijing, China).

Before the biological sensing detection experiment, the microcantilever needed to be pre-processed to remove organic contaminants on the surface of the microcantilever. The pretreatment process of the microcantilever was as follows: First, the microcantilever biosensor was treated with RIE oxygen plasma for 30 s in RF power of 250 W, oxygen flow rate of 20 sccm. Then, the microcantilever biosensor was placed in acetone for 20 min, and this was repeated twice. Subsequently, the microcantilever biosensor was placed in absolute ethanol for 20 min. Finally, the microcantilever biosensor was cleaned with deionized water several times and left to dry naturally in a nitrogen atmosphere. 

For detecting IgG, the microcantilever sensors were constructed by immobilizing a biotinylated goat anti-human IgG on the surface of a sensing microcantilever with antibody concentration of 100 μg/mL by using the biotin streptavidin system (BAS) method. The sensing microcantilever surface functionalization process was as follows:

(a) The pre-processed microcantilever was immersed in 5 mg/mL DDPA for about 1 h to coat the carboxyl groups on the Au film surface of the sensing microcantilever
(b) Then, it was soaked in the EDC/NHS mixture with a concentration of 5 mg/mLs, and a volume ratio of 3:1 for about 30 min to form a succinimide activator on the surface of the sensitive microcantilever.
(c) After being repeatedly cleaned, the microcantilever was immersed in a 0.1 mg/mL streptavidin solution to react for about 1 h to cross-link the streptavidin molecule with the succinimide activator;
(d) At last, a 1 M ethanolamine was injected to inactivate the residual carboxyl groups on the surface of the sensing microcantilever for 30 min after cleaning the microcantilever a few times.

**Figure 8:**
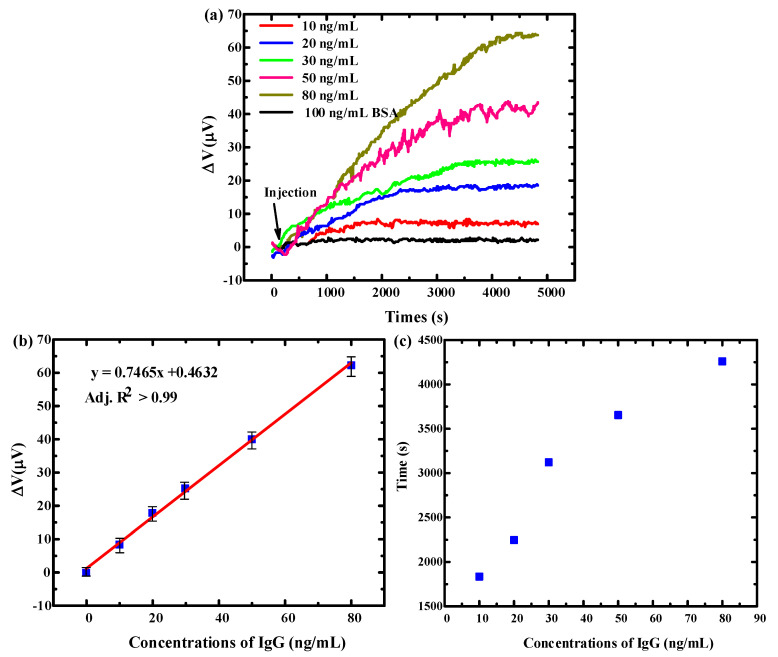
(**a**) The response output curve of PI/Ti/SiO_2_ flexible microcantilever microcantilever to IgG detection (**b**) The linear relationship between the microcantilever response voltage and IgG concentration. (**c**) The relationship between the microcantilever response time and the IgG concentration.

After the functionalization, human IgG with concentrations of 10 ng/mL, 20 ng/mL, 30 ng/mL, 50 ng/mL, and 80 ng/mL were detected with the sensors, the test results are shown in Figure 8a. The relationship between the microcantilever response voltage and IgG concentration is shown in Figure 8b. Within the detection concentration range of IgG, the biosensor showed a good linear response, and its detection limit, the minimum detectable concentration, reached 10 ng/mL. The response time of the sensor is the time when the output voltage signal changed from the injection moment to stable saturation state. The relationship between the microcantilever response time and the IgG concentration is shown in Figure 8c. The response time from injection to dynamic equilibrium were 1835 s, 2246 s, 3120 s, 3654 s, and 4260 s for the concentrations of IgG 10 ng/mL, 20 ng/mL, 30 ng/mL, 50 ng/mL and 80 ng/mL respectively. The limiting factor for response time depends on a complicated dynamic process of immobilized biotinylated goat antihuman IgG and IgG. As the concentration of target IgG increased, the output voltage signal of the sensor becomes larger, and the response time becomes longer. At the same time, the specificity was also tested with 100 ng/mL BSA as a blank control, and the IgG biosensor basically did not respond to it.

### 4.2. Ricin Detection

Ricin is a toxic protein extracted from castor beans. Several traditional analytical techniques for detecting ricin are based on the optical method such as mass spectrometry [29], Raman spectrometry [30] and fluoroimmunoassay [31,32]. A microcantilever aptamer sensor for detecting ricin was constructed by immobilizing a ricin probe on the surface of sensing microcantilever. The DNA sequence of the ricin aptamer is 5′-SH-TCG CAA GAC GGA CAG AAG CTG ATT GTTATT TTT TTT TTT GTT TAT GCT GTA TGC CAT TAG GTT GGT GGA GCG ATT TGT-3′ and was synthesized by solid-phase peptide synthesis and purified until it reached 95% purity by polyacrylamide gel electrophoresis (PAGE) method (AuGCT. LTD., Beijing, China). The thiol modified ricin aptamer was stored at −18 °C for the ricin experiments. Other reagents and materials are the same as mentioned in Section 4.1.

The microcantilever biosensor was cleaned before the functionalization process to remove organic contaminants on the surface of the microcantilever. The cleaning process of the microcantilever biosensor was as follows: First, the microcantilever biosensor was treated with RIE oxygen plasma for 30 s in RF power of 250 W, oxygen flow rate of 20 sccm. Then, the microcantilever biosensor was placed in acetone for 20 min, repeated for twice. Subsequently, the microcantilever biosensor was placed in absolute ethanol for 20 min. Finally, the microcantilever biosensor was cleaned with deionized water several times and dried naturally in a nitrogen atmosphere. 

During the fabrication process, the microcantilever was immersed in a sulfhydryl aptamer solution with a concentration of 20 μM, and incubated for 8 h at room temperature to coat the sulfhydryl aptamer on the surface of the sensitive microcantilever to complete the probe immobilization. After washing the microcantilever sensor chip with PBS solution several times to elute the unfixed and weakly bound aptamer probe, 1% BSA was used to seal the surface of the sensitive beam for about 30 min. Finally, the chip was cleaned several times to elute the unfixed BSA, thus completing the functionalization process of the microcantilever.

**Figure 9:**
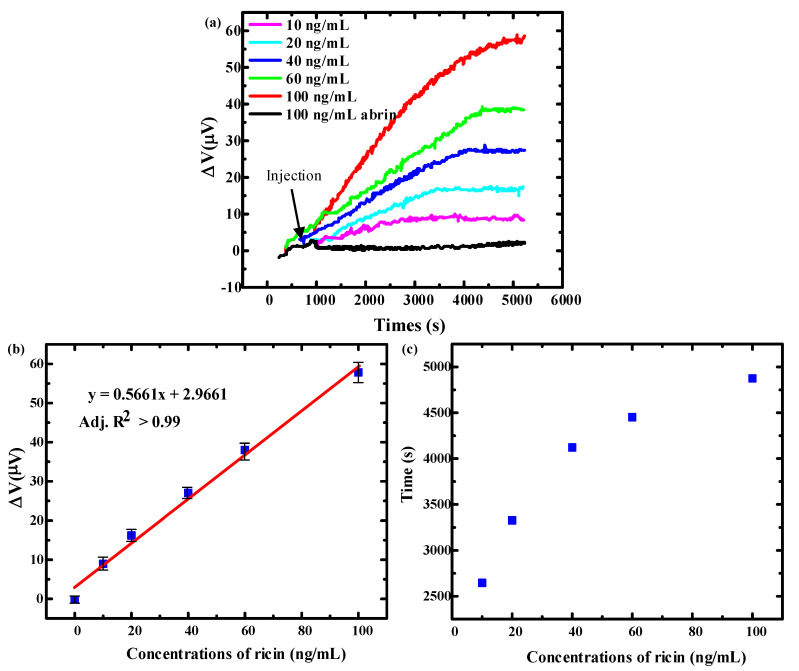
(**a**) The output voltage curve of the microcantilever aptamer sensors at detecting concentration of 10–100 ng/mL ricin (**b**) Linear relationship between microcantilever response voltage and detection concentration. (**c**) The relationship between the microcantilever response time and the ricin concentration.

For the detections, the ricin solution was diluted to 0.2 μg/mL, 0.4 μg/mL, 0.8 μg/mL, 1.2 μg/mL and 2 μg/mL solutions by using the PBS solution beforehand. Firstly, the microcantilever was placed in a reaction well containing 400 μL PBS solution and biased a 3 V voltage until the sensor output signal achieved a stable state. Then, the diluted ricin solutions were injected to the reaction well and to form detection concentrations of 10 ng/mL, 20 ng/mL, 40 ng/mL, 60 ng/mL and 100 ng/mL respectively. The output signals were measured by an Agilent 34,401 A 6½ high-precision multimeter with a sample frequency of 0.5 Hz. Figure 9a shows the sensor’s detection results of ricin at concentrations from 10 ng/mL to 100 ng/mL. The dynamic equilibrium voltage variations were 9 μV, 15 μV, 25 μV, 37 μV, and 55 μV for the concentrations of ricin 10 ng/mL, 20 ng/mL, 40 ng/mL, 60 ng/mL and 100 ng/mL respectively. Figure 9b shows the relationship of microcantilever response voltage and detection concentration; the output voltage of the sensor increases linearly with the concentration of ricin, with the minimum detectable concentration of 10 ng/mL. The relationship between the microcantilever response time and the ricin concentration is shown in Figure 9c. The response time from injection to dynamic equilibrium were 2645 s, 3326 s, 4121 s, 4452 3, and 4875 s for the concentrations of ricin 10 ng/mL, 20 ng/mL, 40 ng/mL, 60 ng/mL and 100 ng/mL, respectively. The limiting factor for response time depend on a complicated dynamic process of the ricin aptamer and ricin [33]. At the same time, abrin, which is also a dimeric glycoprotein, was used as the target molecule to evaluate the specificity of the ricin aptamer sensor. The sensor did not show a significant response to 100 ng/mL abrin, which reflected a good specificity.

## 5. Conclusions

In this work, a low spring constant piezoresistive microcantilever was designed and fabricated using a Ti film as the piezoresistor and a low spring constant PI and a thin SiO_2_ as the top and the bottom insulation layers, respectively. The microcantilever has excellent mechanical properties with 0.02128 N/m spring constant and 1.03 × 10^−7^ nm^−1^ deflection sensitivity. The microcantilever also shows a good electrical stability with less than 3 μV voltage fluctuation in PBS buffer. After surface functionalization with thiol modified ricin aptamer, the microcantilever was introduced to detect ricin with 10 ng/mL minimum detectable concentration and good linear correlation results. All experiment results show that the flexible PI/Ti/SiO_2_ microcantilever prepared in this paper has good biological detection performance and can be used for highly sensitive biomolecule detection.
